# Characterization of Discrete Subpopulations of Progenitor Cells in Traumatic Human Extremity Wounds

**DOI:** 10.1371/journal.pone.0114318

**Published:** 2014-12-09

**Authors:** Geoffrey E. Woodard, Youngmi Ji, Gregory T. Christopherson, Karen M. Wolcott, David J. Hall, Wesley M. Jackson, Leon J. Nesti

**Affiliations:** 1 Department of Surgery, Uniformed Services University of Health Sciences, Bethesda, MD, United States of America; 2 Clinical and Experimental Orthopaedics, National Institute of Arthritis and Musculoskeletal and Skin Diseases, National Institutes of Health, Bethesda, MD, United States of America; 3 Laboratory of Genome Integrity, Nation Cancer Institute, National Institutes of Health, Bethesda, MD, United States of America; 4 Department of Orthopaedic Surgery, Walter Reed National Military Medical Center, Bethesda, MD, United States of America; University of California, San Diego, United States of America

## Abstract

Here we show that distinct subpopulations of cells exist within traumatic human extremity wounds, each having the ability to differentiate into multiple cells types in vitro. A crude cell suspension derived from traumatized muscle was positively sorted for CD29, CD31, CD34, CD56 or CD91. The cell suspension was also simultaneously negatively sorted for either CD45 or CD117 to exclude hematopoietic stem cells. These subpopulations varied in terms their total numbers and their abilities to grow, migrate, differentiate and secrete cytokines. While all five subpopulations demonstrated equal abilities to undergo osteogenesis, they were distinct in their ability to undergo adipogenesis and vascular endotheliogenesis. The most abundant subpopulations were CD29+ and CD34+, which overlapped significantly. The CD29+ and CD34+ cells had the greatest proliferative and migratory capacity while the CD56+ subpopulation produced the highest amounts of TGFß1 and TGFß2. When cultured under endothelial differentiation conditions the CD29+ and CD34+ cells expressed VE-cadherin, Tie2 and CD31, all markers of endothelial cells. These data indicate that while there are multiple cell types within traumatized muscle that have osteogenic differentiation capacity and may contribute to bone formation in post-traumatic heterotopic ossification (HO), the major contributory cell types are CD29+ and CD34+, which demonstrate endothelial progenitor cell characteristics.

## Introduction

The formation of heterotopic ossification (HO) following orthopaedic trauma is a devastating complication that can lead not only to further surgeries but also permanent dysfunction. Clinically significant HO has been observed to develop in approximately 70% of service men and women who sustain a traumatic injury such as a blast wound, which impedes rehabilitation of our wounded veterans [1]–[5]. Though much has been learned of HO in the past decade regarding risk factors, much still remains unknown especially with regard to treatment and prevention. For example, current methods for preventing HO formation may not be appropriate in the acute trauma setting. This is particularly true in instances where there is significant systemic insult, large tissue deficits or fractures, as healing potential could be altered with the use of non-specific treatment regimens [6], [7]. Since any compelling approach to prevention and treatment will depend on knowledge of the basis for which HO forms, it is critical that the cell types and soluble factors be identified within a traumatic extremity wound that lead to HO [8]–[10].

To understand the pathology that underlies HO it is essential that the cell types involved in bone formation be identified [11]. Towards this end it is important to consider the distinct cell populations that already reside within traumatized muscle, a major component of the traumatic extremity wound. A large number of different cell types exist in the soft tissue component of these wounds, which we refer to as traumatized muscle, which could participate directly in bone formation. These types of wounds cross several tissue planes and as such have a heterogeneous cell population that includes, but is not limited to, vascular smooth muscle[12], and vascular endothelial cells [13], myoblasts [14]–[16], satellite cells [17], pericytes [18], Schwann cells [19], neurons [20], monocytes [21], fibrocytes [22]–[24], mesenchymal stem cells [25], [26], fibroblasts [27]–[30] and adipocytes [14], [31]–[33]. While it has been generally speculated that the bone forming cells in HO can be derived from many sources, recent evidence has indicated that vascular endothelial cells (VECs) not only have the capacity and ability to differentiate into osteoblasts in vivo, but in humans and animals with fibrodysplasia ossificans progressive (FOP), VECs originally located in capillaries can be shown to be newly localized to the bony lesions [34], [35]. This data provides compelling evidence that VECs could be the source of bone forming cells in HO [34], [35].

To better understand the cellular contribution to HO, we have sought to identify the most abundant cell types (including VECs) within soft tissue samples obtained from traumatic extremity wounds that have osteogenic capacity, under the assumption that any of these sub-populations could be bone forming candidates. We have generated a initial single cell suspension from these human traumatized muscle wounds and have used this suspension to directly sort the cells by flow cytometry, based on the presence or absence of specific cell surface marker proteins. We have identified multiple distinct cell types within this suspension, each having unique functional characteristics. The cell type that is the most abundant, most proliferatively active, has the highest migration capacity and is capable of undergoing osteogenesis is identified as a likely endothelial progenitor, which could be a major contributor to bone formation in HO and are discussed here.

## Materials and Methods

### Cell Isolation

Soft tissue samples were collected from traumatic extremity wound debridements comprised mostly of injured human muscle from lower extremity wounds sustained as a result of high-energy trauma from Operation Enduring Freedom and Operation Iraqi Freedom. All samples were collected with Institutional Review Board approval at Walter Reed Army Medical Center or Walter Reed National Military Medical Center (G1 90QY). The Walter Reed National Military Medical Center Institutional Review Board waived the need for consent. The protocol for extracting cells from traumatized muscle tissue was based on a modification of previous work (11). Briefly, fat, fascia, other connective tissue, and necrotic tissue were dissected away from the healthy margin of the debrided muscle sample. Approximately 0.5 cc of the remaining tissue was washed three times in Hanks' Balanced Salt Solution (Gibco, Carlsbad, California) and then was extensively minced in a 10-cm culture dish containing Dulbecco's Modified Eagle Medium (Gibco) and 3X (penicillin, streptomycin, Fungizone) (Gibco) until it could pass through the tip of a 25-mL serological pipette (Falcon; BD Biosciences, San Jose, California). The minced tissue was transferred to a 50-mL conical vial with digestion medium containing Dulbecco's Modified Eagle Medium, 3X penicillin/streptomycin/Fungizone, and 0.5 mg/mL collagenase type 2 (Worthington Biochemical, Lakewood, New Jersey). The tissue slurry was agitated gently at 37°C for two hours, and the resulting digest was filtered through a 40-mm cell strainer (Falcon) and centrifuged. At this point if the cells were to be used directly for flow cytometry they were resuspended in PBS plus 0.1% FBS and antibodies as indicated below. If muscle-derived multiprogenitor cells (MPCs) were to be isolated, then the pellet was resuspended in growth medium (Dulbecco's Modified Eagle Medium with 10% fetal bovine serum; Gibco) and 5X penicillin/streptomycin/Fungizone, and then plated onto tissue culture polystyrene (150-cm^2^ flask; Falcon). The cells were incubated at 37°C in a 5% CO2-humidified cell incubator for two hours and then were extensively washed with Hanks' Balanced Salt Solution before fresh growth medium was added with 3X (penicillin, streptomycin, Fungizone). Once MPC colony forming units were observed, the concentration of penicillin/streptomycin/Fungizone was lowered to 1X. Cell confluence was reached after approximately two weeks. The cell cultures were passaged at 1∶4 when confluent.

### FACS analysis, Cell Sorting, Immunophenotyping

For flow cytometry, MPCs or the crude cell suspension derived from muscle (>2×10^5^ cells) were washed and resuspended in phosphate-buffered saline (PBS) + 0.1% FBS (PF), containing titrated concentrations (1∶100 dilution) of the following conjugated mouse IgG1, κ anti-human monoclonal antibodies (BD Biosciences, San Jose, CA): CD14-PE, CD34-PE, CD45-FITC, CD73-PE, CD90-PE, CD105-PE, CD36-FITC, CD91-PE, CD45-FITC, CD167a-PE, CD73-FITC, CD31-PE, CD56-PE, CD117-PE, CD29-FITC, CD34-FITC for 1 h at 4°C. Cell suspensions were washed twice and resuspended in either a FACS buffer for analysis on a Cell Sorter (FACS Aria, BD Biosciences) or in 1% paraformaldehyde for analysis on a flow cytometer (Fortessa, BD Biosciences). Subsequent analyses were performed using the FACS DIVA software (BD Biosciences, San Jose, CA) or FlowJo (Tree Star, Ashland, OR).

### Cell proliferation assay

Freshly pelleted FACS sorted sub-populations of traumatized muscle cells were resuspended in growth medium. Serial dilutions ranging from 50 to 50,000 cells in 200 µl final volumes were prepared in microplates. Six replicate samples were prepared in wells for each serial dilution, along with control (no cells) samples for media background fluorescence measurement, and incubated at 37°C at 5% CO_2_. Every 2 days, 100 µl of growth medium were removed from each well and replaced with 100 µl of fresh growth medium. Microplate cultures were harvested by inverting the microplate onto paper towels with gentle blotting to remove growth medium without disrupting adherent cells. One microplate was prepared for each harvest day. Microplates of sub-population cells were harvested on days 0 through day 15. After harvest, each microplate was kept at −70°C until all microplates had been harvested. All microplates were then thawed at room temperature, and 200 µl of the CyQUANT GR dye/lysis buffer was added to each sample well. The samples were incubated in darkness for 2 to 5 min. Sample fluorescence was measured with a BioTek Synergy H1 Hybrid Multi-Mode Microplate Reader, and growth curves were plotted as cell number per well versus time.

### Cell Migration - Zone exclusion assay

The rate of traumatized muscle cell sub-population migration was determined using a 2D gap-closure radius in a 96-well migration assay, according to manufacturer's instructions (Cell Biolabs, San Diego, CA, USA). Migration was evaluated up to 36 hrs in replicates (N = 6). Cells were stained with a non-toxic green fluorescent metabolic dye, Calcein AM for 10 min. at 37°C at each time interval (Cat. 80011-2, Biotium, Hayward, CA). Afterwards, photographs were taken with a Nikon digital camera attached to a Nikon Eclipse T*i* inverted microscope and gap-closure was measured.

### ELISA Cytokine assay

Cell culture supernatants were used for enzyme-linked immunosorbent assay (ELISA) experiments. Supernatants of primary cells after 48 h in culture were concentrated using Amicon Ultra centrifugal 3,000 molecular weight cut-off (MWCO) filters (Ultracel 3K; Millipore, Billerica, MA, USA). Cell culture media supernatant was diluted 1∶10 before being loaded into the wells. Sandwich ELISAs were prepared using the TGFß1, TGFß2, TGFß3, and VEGF Duo Kits (R&D Systems, Minneapolis, MN, USA). Direct ELISAs were performed using Laminin1 and CTGF monoclonal antibodies (Sigma, St. Louis, MO, USA). The ELISA procedure was carried out following the recommended instructions. All analyses and calibrations were performed in replicate (N = 8).

### Osteogenesis, adipogenesis and vascular endothelial differentiation

Cells were induced to undergo adipogenic and osteogenic differentiation, as described previously [25], [36]. Briefly, for adipogenic differentiation, cells were seeded into six-well tissue-culture plates at a density of 20,000 cells/cm^2^ and treated for 3 weeks with adipogenic medium, consisting of DMEM with 10% FBS, and supplemented with 0.5 mmol/L 3-isobutyl-1-methylxanthine (IBMX), 1 µg/ml insulin, and 1 µmol/L dexamethasone (Sigma). For osteogenic differentiation, cells were seeded into six-well plates (Corning) at a density of 20,000 cells/cm^2^, and treated for 3 weeks with osteogenic medium, consisting of DMEM with 10% FBS, and supplemented with 10 mmol/L ß-glycerolphosphate, 10 nmol/L dexamethasone, 50 µg/ml ascorbic acid-2-phosphate, and 10 nmol/L 1,25 dihydroxyvitamin D_3_ (Biomol International L.P., Plymouth Meeting, PA).

For differentiation into vascular endothelial cells, the subpopulations were seeded into 24-well plates (Corning) at a density of 5,000 cells/cm^2^. The cells were then treated for 2 weeks with endothelial progenitor cell medium (Promocell), consisting of 10% FBS, and supplemented with 50 ng/ml VEGF (Promocell) or were treated with growth media as a control. Fluorescence microscopy utilizing antibodies against the endothelial makers Tie2, VE-Cadherin and CD31, was used to identify vascular endothelial cells.

### Oil Red O and Alizarin Red Staining

Three-week adipogenic cultures were rinsed twice with PBS, fixed in 4% buffered paraformaldehyde, stained with Oil red O (Sigma) for 5 minutes at room temperature, and counterstained with Harris-Hematoxylin Solution (Sigma), to visualize lipid droplets. Three week osteogenic cultures were fixed with 60% isopropyl alcohol and stained for 3 minutes with 2% (wt/vol) Alizarin Red S (Rowley) at pH 4.2 to detect mineralization.

### Statistical Analysis

Statistical significance was determined using Student's t-test or the Mann-Whitney U test. *P*<0.05 was considered significant. The data are expressed as the mean ± standard error of the mean (SEM) with the sample size (n value).

## Results

**Figure 1:**
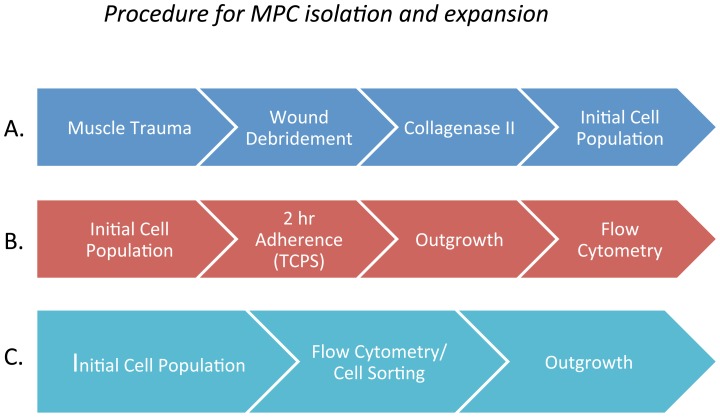
Outline of the procedure for isolating progenitor cells from muscle. Figures 1A and 1B. Previously used procedure that entailed a 2 hour adherence to tissue culture polystyrene (TCPS) following by a 2 week (wk) outgrowth in tissue culture media followed by a flow cytometry analysis. Figures 1A and 1C. Procedure used in this study. A single cell suspension was used directly for flow cytometry analysis and cell sorting.

**Figure 2:**
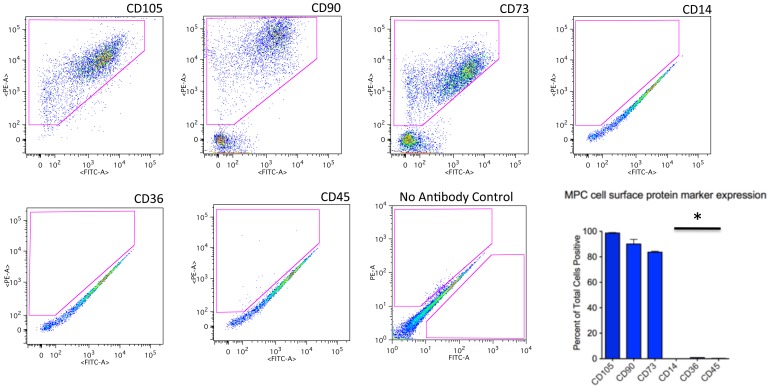
Flow cytometry analysis of cells isolated from traumatized muscle by 2-hour adherence to TCPS. Cells were isolated and cultured after 2-hr adherence to TCPS (Figure 1B). The cells were then processed for flow cytometry using fluorescent-conjugated antibodies against the cell surface markers indicated in the figure. The gated regions, indicated by a red geometric figure within the plot, containositive cells. Also shown is a bar graph reflecting the number of positive cells for each marker (mean ± SEM, n = 8). Significant difference is noted (*) between the CD14, CD36, CD45 as compared to CD105, CD90, CD73 (Mann Whitney U Test, p≤0.01).

To identify the cells within traumatized muscle that might contribute to HO formation, we derived a crude cell population from debrided and collagenase II-treated traumatized human muscle tissue by the scheme outlined in Fig. 1A [14]. We have used a two-hour adhesion kinetics based method of cell harvesting on tissue culture plastic (TCPS) to isolate multiprogenitor cells (MPCs) from this traumatized muscle cell preparation (Fig. 1B) [14]. Following culture and expansion of these cells we have identified three marker proteins that were expressed on these MPCs; CD73, CD90 and CD10 [14], [25], [37]. As shown in Fig. 2, 66%, 71% and 97% of the adherent cells are positive for CD73, CD90 and CD105 respectively. The MPCs were essentially negative for CD14, CD36 and CD45, which are markers for hematopoietic cells (Fig. 2). That these cells are positive for CD73, CD90 and CD105 suggests that they have mesenchymal characteristics [38].

**Figure 3:**
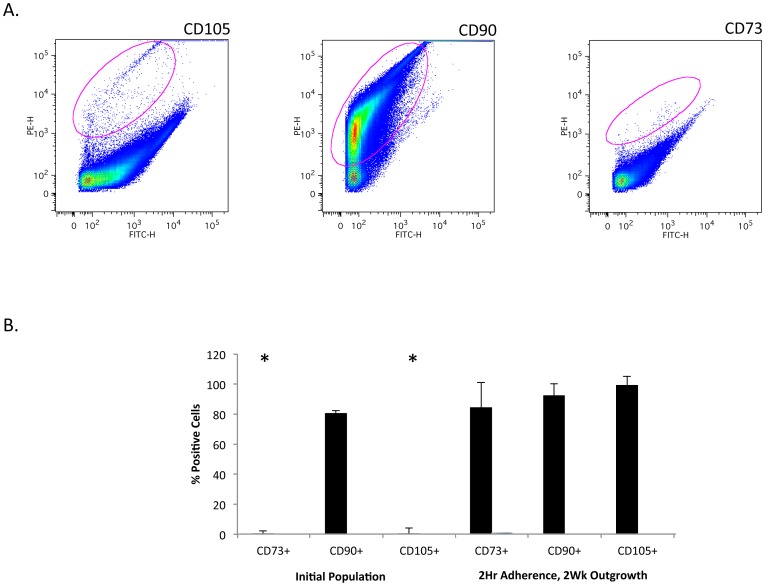
Analysis of CD105, CD90 and CD73 positive cells in a single cell suspension from traumatized muscle. A. single cell suspension (Figure 1C) from collagenase digested traumatized muscle tissue was processed for flow cytometry using fluorescent-conjugated antibodies against the cell surface markers indicated in the figure. The gated regions, indicated by a red circle within the plot, contain the positive cells. B. For each marker the bar graph reflects the number of positive cells isolated (mean ± SEM, n = 12) as in Figure 1B (2 Hr Adherence, 2 Wk outgrowth) or as in Figure 1C (Initial Population). Significant difference is noted (*) between CD73 and CD105 in the initial population and the other conditions (Student's t-test, p≤0.01).

To gain greater insight into the cells residing within muscle *in vivo* it was important to assess marker protein expression on cells within the initial crude cell population, before they were cultured and expanded as outlined in Fig. 1C. When we perform cell sorting on the initial population, we found that CD73 and CD105 are significantly reduced in these cells while CD90 was abundant (Fig. 3A). When a comparison is made between the initial cell population and the cells that have been expanded in culture it is clear that expression of CD73 and CD105 arises during outgrowth of cells (Fig. 3B). This data suggest that these markers may be inappropriate for use in identifying cell types within the original traumatized muscle tissue. Since CD73 and CD105 are considered markers for mesenchymal stem cells the data suggests that as these cells are cultured they undergo differentiation down into a mesenchymal lineage [38].

It was therefore essential that additional cell surface markers be employed to attempt to identify subpopulations of cells within traumatized muscle that would contribute to HO. A list of these markers is shown in Table 1. The rationale for the choice of these markers is based on data indicating that they generally identify discreet sets of cells within a tissue. The marker protein function and known sites of expression are listed in Table 1. The goal was to select for individually enriched CD31, CD34, CD56, CD91 and CD29 positive cells (markers for endothelial, nerve and muscle cells as outlined in the Table 1) that were individually depleted for CD45 or CD117, which are primarily expressed on hematopoietic cells and T-cells.

**Table 1:** Markers Used to Sort Subpopulations from Traumatized Human Muscle.

Antibody	Protein Function	Sites of Expression
CD29	Integrin Beta 1	Widely expressed, skeletal muscle, mesenchymal stem cells
CD31	Platelet/Endothelial Cell Adhesion Mol (PECAM)	Platelets and endothelial cells
CD34	Sialomucin	Endothelial cells, endothelial progenitor cells, hematopoietic progenitor cells
CD45	Protein Tyrosine Phosphatase RC (PTPRC)	T-cells, B-cells, hematopoietic stems cells
CD56	Neural Cell Adhesion Molecule (NCAM)	Neurons, T cells, myoblasts, satellite cells (quiescent & proliferating)
CD91	Low Density Lipoprotein Receptor-Related Prot (LRP1)	Vascular smooth muscle, myoblasts, endothelial cells, monocytes, neurons

**Figure 4:**
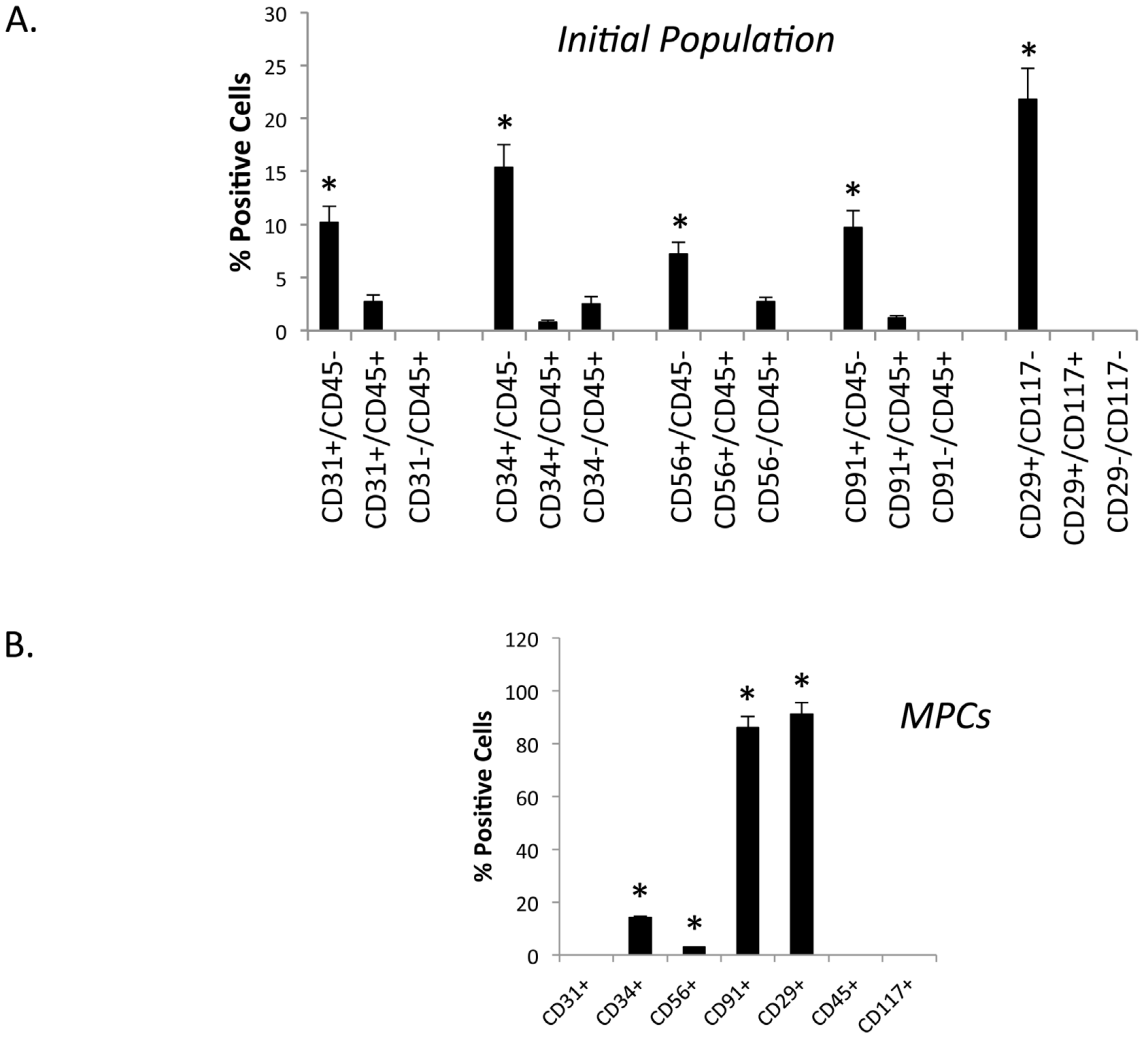
Flow cytometry analysis of a single cell suspension from traumatized muscle. A. A single cell suspension from collagenase digested muscle tissue (Figure 1C) analyzed directly by flow cytometry using fluorescent-conjugated antibodies against the cell surface markers indicated in the figure. The bar graph indicates the number of positive cells for each marker (mean ± SEM, n = 6). Significant difference is observed (*) between the first sub-population compared to the next two sub-populations for all five sets (Mann Whitney U test, p≤0.01). B. Cells isolated after two hours adherence, (Figure 1B) were analyzed by flow cytometry using the indicated antibodies. The bar graph indicates the number of positive cells for each marker (mean ± SEM, n = 8). Significant difference is observed (*) between CD34, CD56, CD91, CD29 and CD31, CD45 and CD117 (Mann Whitney U test, p≤0.01).

Flow cytometry analysis was performed on the initial population using combinations of the above antibodies. The outcome of cell sorting of the initial crude cell population from traumatized muscle is shown in Fig. 4A. As is evident from Fig. 4A, each subpopulation was present in the range of 7.5% (CD56+/CD45−) to 21.8% (CD29+/CD117−). The CD31+, CD34+ and CD91+ subpopulations varied from 10% to 15%. The CD45+ cells comprise 2% or less of the total population, and the CD117+ cells were present at much less than 1%. Since both of these marker proteins are predominantly expressed on hematopoietic cells and the populations were less than 2% it was decided not to pursue them in this study.

In contrast to the freshly isolated cells from traumatized muscle, when the MPCs (cells isolated after a 2-hour adherence to TCPS) were analyzed by flow cytometry, over 90% were positive for CD29 and CD91 (Fig. 4B). Fourteen percent were positive for CD34, 3 percent were positive for CD56 and less than 1% was positive for CD31. Thus it appears that the two-hour adherence selection and outgrowth of MPCs leads to either elevated expression of CD29 and CD91 or to an enriched selection of CD29 and CD91 positive MPC cells, without a corresponding elevation in CD34 positive MPC cells. Further, CD56 and CD31 levels were very low indicating they may be selected against in this isolation process.

To next determine if the most abundant subpopulations from the initial cell isolation shown in Fig. 4A were actually distinct cell populations, we analyzed the initial cell isolate for three selection markers by flow cytometry. This was done to determine if for example the CD34+ cells overlapped with the CD31+ cells. As can be seen in Table 2, there was significant overlap between the CD34+ and the CD29+ cells. 99.2% of the CD34+ were also CD29+, while 82% of the CD29+ cells were CD34+. However, we did not observe any significant overlap between any of the other markers. All were less than 1%. These data indicate that CD34, CD31, CD56 and CD91 identify four distinct subpopulations within traumatized muscle. These four subpopulations accounted for roughly 43% of the total initial cell population from traumatized muscle. Thus by the process of elimination, the most likely target cell types for these markers from traumatized muscle are endothelial progenitor cells.

**Table 2:** Three-way FACS Analyses Identifies Likely Targets of Markers Used to Sort.

3-Way Marker Combination	% Positive Cells
CD34+/CD29+/CD45−	15%
CD34+/CD31+/CD45−	<1%
CD34+/CD56+/CD45−	<1%
CD34+/CD91+/CD45−	<1%
CD31+/CD56+/CD45−	<1%
CD91+/CD56+/CD45−	<1%
CD31+/CD91+/CD45−	<1%

Subpopulations From Traumatized Human Muscle.

### Proliferation/Migration

Next these subpopulations were tested for functional differences in terms of proliferative capacity, migration ability, secretion of inflammatory cytokines as well as osteogenesis, adipogenesis and vascular endothelial cell differentiation. To test for these characteristics, the cell types from Fig. 4 (i.e. CD31+, CD34+, CD56+, CD91+, CD29+) were first sorted and subcultured and then the functional assays were performed.

**Figure 5:**
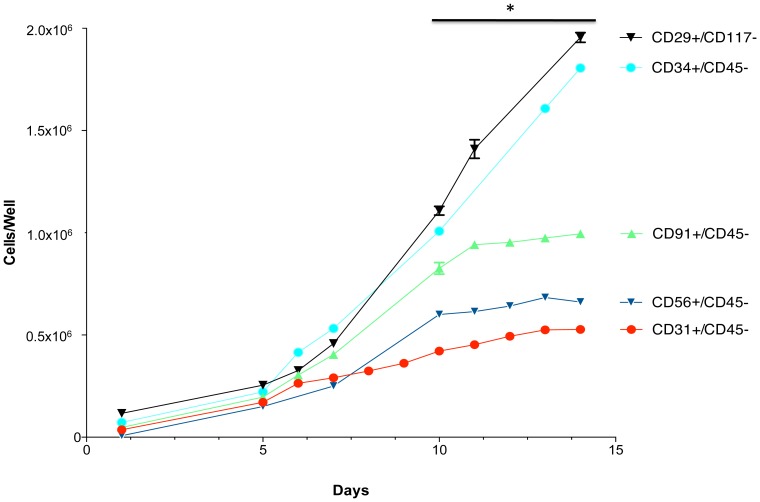
Growth characteristics of CD29, CD31, CD34, CD56 and CD91 positive cells. Flourescent-conjugated antibodies to CD29, CD31, CD34, CD56 and CD91 were used to separately sort and subculture cells form collagenase 2 digested human muscle. The cells were seeded at 1×10^5^ cells per well in 6-well plates on day 0 and cultured for an additional two weeks. The graph indicates the cell number on the indicated days (mean ± SEM, n = 8). A significant difference is noted (*) between cell growth number for replicates between 10 and 15 days of growth for all five cell populations (Mann Whitney U test, p≤0.01).

When growth rates were assessed, as shown in Fig. 5, it was clear that over a two-week period marked differences exist between the cell types in terms of proliferative capacity. The CD31 and CD56 populations grew very slowly while the CD91 cells grew only moderately well. The CD34 and CD29 cells grew equally well and significantly better than the other cell types (e.g. approximately three times faster than the CD31+ and CD56+ cells). These data suggest that the CD34+ and CD29+ cells are the most proliferative cell types isolated from traumatized muscle based on proliferation rates *in vitro* [25].

**Figure 6:**
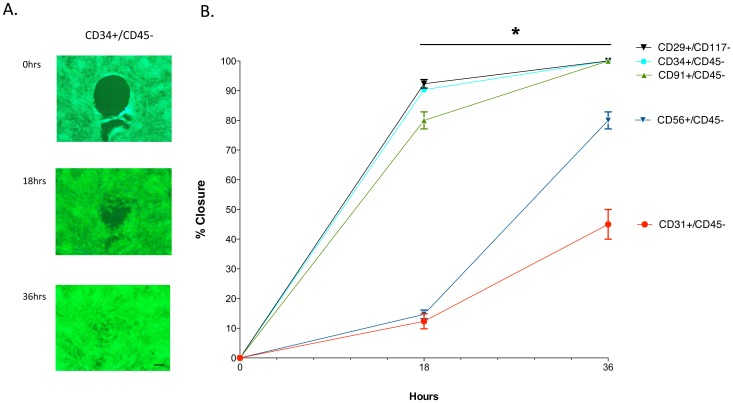
Migration characteristics of CD29, CD31, CD34, CD56 and CD91 positive cells. Fluorescent-conjugated antibodies to CD29, CD31, CD34, CD56 and CD91 were used to separately sort and subculture cells from collagenase 2 digested human muscle tissue. The cells were seeded into wells of a Gap-Closure Assay plate. When the wells were confluent the circular plug was removed to allow the cells to migrate into the space. The graph indicates the % closure of the circular space at the indicated times (mean ± SEM, n = 8). A significant difference is noted (*) in migration rates between CD56 and CD31 compared to CD29, CD34 and CD91, between time points of 18 to 36 hours (Mann-Whitney U Test, p≤0.01). Scale bar  = 100 µm.

A counterpart to proliferation is migration, or the ability of cells to fill in a wound bed. A gap-closure assay was performed on the subpopulations as shown in Fig. 6A for the CD34+ cells, where the circular excluded zone is slowly filled in by cell migration over a 36-hour period. When all the subpopulations were examined using this assay, it was apparent from Fig. 6B that the CD34+ and CD29+ cells along with the CD91+ cells had the highest migratory ability. The CD31+ and CD56+ cells demonstrated a significantly slower migratory ability.

### Inflammatory Cytokine Secretion

**Figure 7:**
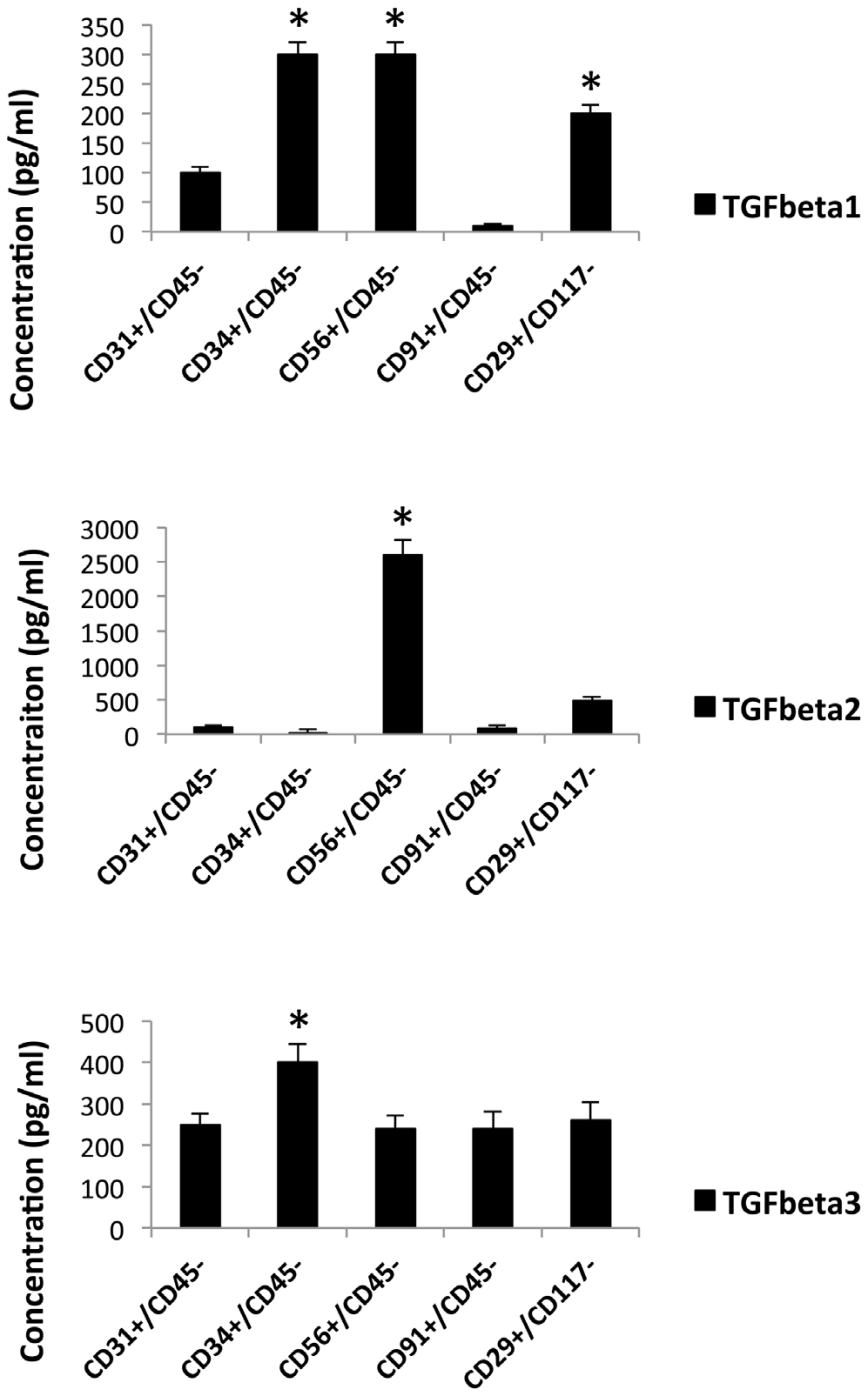
Inflammatory cytokine secretion of CD29, CD31, CD34, CD56 and CD91 positive cells. Flourescent-conjugated antibodies to CD29, CD31, CD34, CD56 and CD91 were used to separately sort and subculture cells form collagenase 2 digested human muscle tissue. The cells were cultured to confluence and the supernatants were removed and assessed for TGFß1, TGFß2 and TGFß3 levels by ELISA. The graph indicates the concentrations of the cytokines (mean ± SEM, n = 8). A significant difference in amounts (mean pg/ml) is noted (*) between CD34, CD56 and CD29 for TGFß1, CD56 for TGFß2 and CD34 for TGFß3 when compared to the other populations (Mann-Whitney U Test, p≤0.01).

It has been previously demonstrated that members of the TGFß family are expressed to high levels in traumatized muscle [39], [40]. Since these inflammatory cytokines are thought to mediate much of the wound-healing and fibrotic response it was important to determine if a subpopulation of cells within the wound is responsible for expression of a critical TGFß family member. The five subpopulations were therefore assessed for production of TGFß1, TGFß2 and TGFß3 by Elisa assay and the results are shown in Fig. 7A, B and C respectively. From the data it is clear that there are significant differences in the protein expression profile between the different subpopulations with regard to secretion of TGFß1 and TGFß2. CD34+, CD56+ and CD29+ demonstrated high levels of TGFß1 expression while CD31+ had only moderate levels. CD91+ cells produced only low levels of TGFß1.

Surprisingly, as shown in Fig. 7B, the CD56+ cells demonstrated very high levels of TGFß2 expression while the CD29+ cells expressed only moderate amounts. The other three subpopulations produced low levels of TGFß2. And finally, there was no appreciable difference between the subpopulations in terms of expression of TGFß3 (Fig. 7C). In total these data show that these subpopulations vary widely in their production of two members of the TGFß family.

### Osteogenesis and Adipogenesis

**Figure 8:**
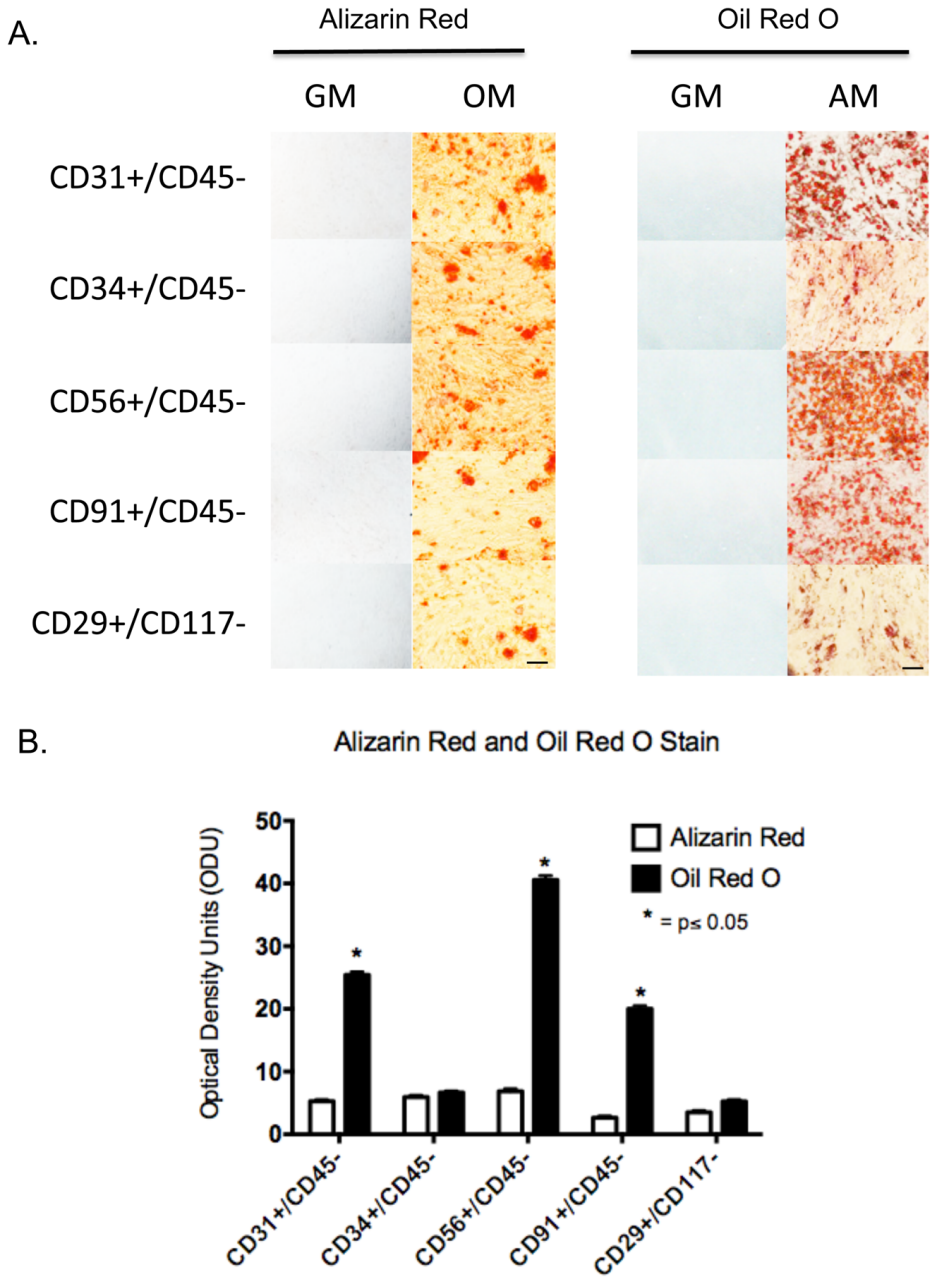
Osteogenesis and adipogenesis of CD29+, CD31+, CD34+, CD56+ and CD91+ cells. A. Flourescent-conjugated antibodies to CD29, CD31, CD34, CD56 and CD91 were used to separately sort and subculture cells form collagenase 2 digested human muscle. The cells were cultured to confluence then the cells were cultured for an additional three weeks in either osteogenic media (OM), adipogenic media (AM) or regular growth media (GM). The cultures were fixed and processed for Alizarin Red or Oil Red O staining. Scale bar  = 100 µm. B. For each marker the bar graph reflects the number of optical density units (ODU) (mean ± SEM, n = 5), as measured with Image J, for Alizarin and Oil Red O staining of isolated cell types differentiated in osteogenic media (OM) and adipogenic media (AM). Significant difference is observed (*) in Oil Red O staining between CD34 and CD29 compared to CD31, CD56, and CD91 (Student's t-test, p≤0.05).

From the data above it seems clear that these subpopulations differ in their abilities to proliferate, migrate and produce inflammatory cytokines; therefore, it may be expected that their abilities to differentiate may also be distinct. The subpopulations were monitored for their capacity to undergo osteogenesis and adipogenesis. As shown in Fig. 8, all five subpopulations displayed similar extents of mineralization after three weeks in osteogenic media, as shown by equal Alizarin Red staining. However, when adipogenesis was assessed, by Oil Red O staining, it appeared that the CD31+, CD56+ and CD91+ cells were approximately three to four fold better able to accumulate lipid than the CD34+ and CD29+ cells. These data indicate that while there were likely no differences in osteogenesis between the cell types, the subpopulations display significant differences in adipogenesis.

### Vascular Endothelial Differentiation

**Figure 9:**
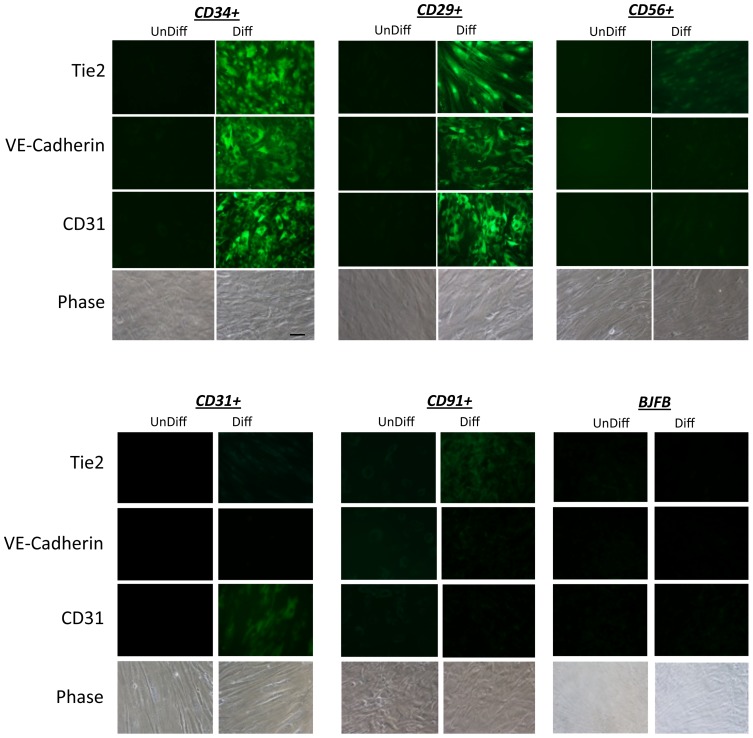
Endothelial cell differentiation of CD29+ and CD34+ cells. Flourescent-conjugated antibodies to CD29, CD31, CD34, CD56 and CD91 were used to separately sort and subculture cells form collagenase 2 digested human muscle. The cells were cultured to confluence then were cultured for an additional two weeks in either vascular endothelial cell differentiation media (VEM) or regular growth media (GM). The cultures were fixed and processed for immunofluorescence microscopy using antibodies to VE-cadherin, Tie2 and CD31. Also shown is a representative phase contrast image of each cell type. Scale bar  = 50 µm.

Since CD34 is a marker of endothelial progenitor cells [36], [40]–[43], the CD34+ cells should have the capacity to differentiate into vascular endothelial cells (VECs) under the appropriate conditions. To determine if is the case, all five subpopulations were cultured for two weeks in endothelial cell differentiation media. As shown in the fluorescence microscopy images in Fig. 9, the CD34+ and the CD29+ cells expressed high levels of the VEC markers CD31, VE-Cadherin and Tie2, but only when cultured in differentiation media. In normal growth media these markers were not expressed. In contrast the CD31+, CD56+ and CD91+ subpopulations did not express detectable levels of these VEC marker proteins, with exception of CD31+ cells, which expressed low levels of CD31 as expected. These data indicate that the CD34+ and CD29+ cells displayed characteristics of a vascular endothelial progenitor cell type.

## Discussion

Here we identify at least four distinct subpopulations within an initial suspension of cells derived from traumatized human muscle. Our initial selection yields cell populations defined by CD31+/CD45−, CD34+/CD45−, CD56+/CD45−, CD91+/CD45− and CD29+/CD117−. By selecting against CD45 and CD117 in these subpopulations we eliminated most hematopoietic lineage cells from our cell sorting. Since the CD34+/CD45− and CD29+/CD117− populations appear to overlap significantly (between 82 to 99%) they will hereafter simply be referred to as CD34+/CD29+ cells. Combined, these four subpopulations (i.e. CD34+/CD29+, CD31+, CD56+ and CD91+) make up approximately 43% of the total cells initially harvested from the traumatized tissue. All four subpopulations are found to be equally capable of mineralizing under osteogenic culture conditions, so it is therefore possible that any or all of these subpopulations could contribute to bone formation in HO. However, given the characteristics of the CD34+/CD29+ cells, it is possible that they are a major cellular contributor to bone formation, as reasoned below.

CD34 is a widely used as a marker of vascular endothelial cells [44], vascular endothelial progenitor cells [34], [45], [46], hematopoietic stem cells [46] and murine satellite cells [47]–[49]. While CD34 is a marker for such different cells the data presented here would lead one to the conclusion that CD34 identifies a vascular endothelial progenitor cell type in traumatized muscle. First, hematopoietic stem cells can be ruled out since we select against CD45 and CD117. Second, the CD34+ and CD29+ cells do not express markers of endothelial cells but could be induced to express them (i.e. CD31, Tie2 and VE-cadherin) in an endothelial differentiation assay. This makes it unlikely that the CD34+ cells are mature endothelial cells or satellite cells, which do not appear to be CD34+ [50]. Third, the CD34 and CD29 cells make up about a 17% of the initial population, a much higher number than that for satellite cells which are usually around 2–7% of the cells in muscle [51]. Fourth, the CD34+ cells appear much larger than satellite cells, which begin as rounded cells that become long and spindle-shaped in culture [52]. Finally, human satellite cells have never been demonstrated to be positive for CD29 and CD34 but have for CD29 and CD56 [47]–[49]. These data make it most likely that the CD34+ and CD29+ subpopulation is a vascular endothelial progenitor cell type (VEPC). If they are scattered throughout the vasculature and activated upon injury then they could be easily recruited to repair injured blood vessels [53].

Given that VECs have been demonstrated to contribute to bone formation in FOP (8), a disease related to HO, it seems probable that vascular endothelial progenitor cells (VEPCs) would also be critical to this process. Since VEPCs would contribute directly to the formation VECs, which could go on to form bone, this would then make VEPCs a primary driver of bone formation in FOP and by extension HO. A hypothesis to be drawn from this data is that the number of VEPCs and the level of osteogenic signals within a wound directly determine the extent of HO that would be formed within the muscle. It could then be predicted that in the absence of CD34 (e.g. CD34^−/−^ mice), HO would either not form or form less efficiently following traumatic injury. Efforts are currently underway to determine if this is the case. Additionally efforts are underway to identify the osteogenic signals that lead to bone formation, a prime candidate being TGFß family members (13,14).

With regard to the other cell types identified here (i.e. CD31+, CD56+, CD91+), while they are distinct from the CD34+ subpopulation, they nonetheless have the capability to mineralize and all could potentially contribute to bone formation in HO. Further, the CD56+ cells are of interest since they secrete high levels of both TGFß1 and TGFß2, mediators of both wound healing and the endothelia/mesenchymal transition [35]. These CD56+ cells could therefore provide both autocrine and paracrine signals necessary for conversion of a variety of cells within muscle to a mesenchymal lineage prior to undergoing osteogenesis. Additionally, the CD56+ subpopulation has been linked to bone formation in HO since it has recently been shown that cells positive for this marker can be isolated from muscle and can mineralize in culture [11], [54]. Similarly, the Wagers laboratory was able to demonstrate osteogenic and adipogenic potential in fetal human myofiber-associated (hMFA) cells that were positive for CD29 and CD34 but variable levels of myogenicity and osteogenicity for CD56 positive cells [47]. The CD31+ subpopulation on the other hand may represent a more mature endothelia cell immediately following isolation, which could have lost the other markers of endothelial cells during subculture (i.e. Tie2 and VE-Cadherin). Finally, regarding the CD91+ subpopulation, it is known that this marker (LRP1) is expressed in a number of cells types such as monocytes/macrophages, neurons, endothelial cells and vascular smooth muscle. However, given our selection protocol the most likely cell type identified by this marker in our selection may be vascular smooth muscle cells [55] which are known to undergo osteogenesis under defined conditions [56], [57].

In conclusion it appears that as may be expected, multiple cell types exist within traumatized muscle, each having the capacity to undergo mineralization *in vitro*. While all may contribute to bone formation in HO some may do so by secreting critical growth factors (e.g. CD56+) while others such as the CD34+/CD29+ may contribute most to mineralizing simply due to an overabundance of cells. Current efforts are directed at determining if the CD34+/CD29+ cells are indeed the most abundant cell type within the wounded muscle as may be hypothesized from the data presented here. Efforts are also underway to explore the roles of these specific cell types more deeply in wound healing of traumatized human muscle.
